# Timing of preemptive vascular access placement: do we understand the natural history of advanced CKD?: an observational study

**DOI:** 10.1186/1471-2369-14-115

**Published:** 2013-05-28

**Authors:** Nisha Bansal, Chenyin He, Daniel P Murphy, Kirsten L Johansen, Chi-yuan Hsu

**Affiliations:** 1 Division of Nephrology, University of California, San Francisco, 521 Parnassus Ave, Box 0532, San Francisco, CA, 94143, USA

**Keywords:** Advanced CKD, AVF, Dialysis, ESRD, Progression

## Abstract

**Background:**

Little is known about the targets and expectations of practicing nephrologists with regard to timing of preemptive AV access surgery and how these relate to actual observed practice patterns in clinical care.

**Methods:**

We administered a 8-question survey to assess nephrologists’ expectations for preemptive vascular access placement to 53 practicing nephrologists in California. We performed a retrospective chart review of 116 patients who underwent preemptive vascular access placement at a large academic medical center and examined progression to ESRD.

**Results:**

According to our survey of nephrologists, most aimed to have preemptive vascular access created about 6 months prior to start of ESRD or when the chances of ESRD within the next year is two-thirds or greater. The estimated GFR level at which they believe match these conditions is approximately 18 ml/min/1.73 m^2^. Among the 116 patients with CKD who underwent preemptive vascular access creation, the mean estimated GFR at the time of access creation was 16.1 (6.8) ml/min/1.73 m^2^. Only 57 out of the 116 patients (49.1%) patients initiated maintenance HD within 1 year after surgery.

**Conclusions:**

In our study, most nephrologists aim for preemptive vascular access surgery approximately 6 months prior to the start of HD. However in fact, only approximately 50% of patients who underwent preemptive vascular access surgery started HD within 1 year. Better tools are needed to predict the natural history of advanced CKD.

## Background

Optimal education regarding different renal replacement modalities and timely preparation for end-stage renal disease (ESRD) treatment are widely accepted to be among most important benefits conferred by early referral of chronic kidney disease (CKD) patients to nephrologists. For patients who are not candidates for preemptive kidney transplant and who elect eventual hemodialysis (HD), a cornerstone intervention is preemptive surgery for arteriovenous (AV) fistula. Numerous studies have shown that incident ESRD patients who initiate HD with a functioning AV fistula fare better, especially compared with their counterparts who initiate HD with a tunneled catheter [1,2]. National initiatives such as Fistula First promote preemptive creation of AV fistulae [3].

Unfortunately, existing guidelines regarding optimal timing for preemptive vascular access creation are not based on high quality evidence and provide conflicting recommendations [4]. For example, the latest Society of Vascular Surgery guidelines recommend that “patients with advanced CKD disease (late stage 4, MDRD < 20-25 mL/min) who have elected HD as their choice of renal replacement therapy be referred to an access surgeon in order to evaluate and plan construction of AV access…(…very low-quality evidence)” [5]. The Canadian Society of Nephrology urge establishment of “AV fistulae when the patient has an estimated GFR of 15 to 20 ml/min and progressive kidney disease (Grade D, opinion)” [6,7]. The 2001 National Kidney Foundation KDOQI guidelines stated that patient “with chronic kidney disease should be referred for surgery to attempt construction of a primary AV fistula when their creatinine clearance is < 25 mL/min, their serum creatinine level is >4 mg/dL, or within 1 year of an anticipated need for HD… (Opinion)” [8]. This was subsequently revised in 2006 without evidential justification to: “A fistula should be placed at least 6 months before the anticipated start of HD treatments. This timing allows for access evaluation and additional time for revision to ensure a working fistula is available at initiation of HD therapy (B)” [9].

In an ideal world, guidelines regarding optimal timing of AV access surgery and physicians’ recommendation to patients in this arena should be grounded in a thorough understanding of the natural history of advanced CKD. For example, if the goal is for patients to undergo access surgery 6 or 12 months prior to anticipated start of maintenance dialysis, what should the corresponding (estimated) glomerular filtration rate (GFR) threshold be? Similarly, what is the likelihood of ESRD at a given GFR level? To better understand these issues, we surveyed nephrologists to better understand physician expectations regarding preemptive AV access surgery. Then we examined current preemptive AV fistula practice pattern at an academic medical center and estimated, using a newly developed equation, the likelihood of ESRD among patients who actually underwent AV access surgery.

## Methods

### Survey of nephrologists

To explore clinical considerations and thresholds for vascular access referral, we administered a voluntary and anonymous short 8-question survey (Additional file 1) to three populations of physicians: 1) full time academic faculty members based at one of the three main University of California at San Francisco (UCSF) teaching hospitals: UCSF Medical Center, San Francisco Veterans Affairs Medical Center and San Francisco General Hospital; 2) community and academic nephrologists attending a dinner presentation in San Diego in September, 2012 and 3) community and academic nephrologists attending an annual medical directors’ meeting for a non-profit dialysis company in September 2012. We excluded current fellows in training. We collected demographic information about the survey participants. Survey questions included: How many months prior to the initiation of HD should an AV fistula be created? At what estimated GFR should an AV fistula be created? At what likelihood of ESRD within 1 year should an AV fistula be created? (Additional file 1).

### Current preemptive vascular access creation practice pattern

**Figure 1:**
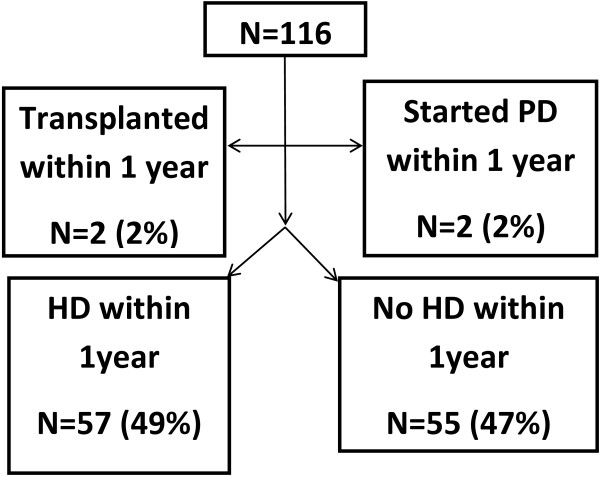
Outcomes after preemptive vascular access placement among 116 adults with advanced chronic kidney disease.

Medical records for all consecutive patients seen by nine attending physicians at the University of California at San Francisco (UCSF) Medical Center Nephrology and Hypertension Faculty Practices between July 1, 1999 and December 31, 2010 were reviewed (N = 2995). We identified 122 patients who underwent vascular access creation (either AV fistula or AV graft). Of the 122, 6 were excluded since they did not have follow-up information within 1 year of vascular access placement, leaving a final analytic sample of N = 116 (Figure 1). Information was abstracted from clinic notes and electronic medical records including: demographics, comorbidities and estimated GFR (by the Modification of Diet in Renal Disease study equation) [10]. All patients were followed for at least one year following surgery.

### Probability of ESRD according to prediction equation

As noted above, current guidelines mostly rely on a static (estimated) GFR threshold or anticipated time to ESRD to guide optimal timing of preemptive vascular access surgery. It is possible that an alternative risk-score based approach may be superior (i.e. preemptive vascular access surgery will be recommended only for patients whose likelihood of developing ESRD in the near future exceeds a certain threshold). To explore this, we estimated probability of ESRD using a recently published predictive model by Tangri et al., among all 116 UCSF Medical Center patients who underwent preemptive vascular access placement [11].

Institutional review board approval from the UCSF Human Research Protection Program was obtained for this study.

## Results

### Surveys of nephrologists

The results of the 3 surveys were remarkably consistent (Table 1). Most nephrologists aimed to have preemptive vascular access created about 6 months prior to start of ESRD or when the chances of ESRD within the next year is two-thirds or greater. The estimated GFR level at which they believe match these conditions is approximately 18 ml/min/1.73 m^2^.

**Table 1:** Survey of practicing nephrologists on timing of preemptive vascular access placement

	**UCSF Nephrologists**	**San Diego nephrologists**	**Medical directors**
	**(N = 16)**	**(N = 8)**	**(N = 29)**
**Survey participant characteristics**			
Age, years, mean(SD)	46.1 (13.0)	43.1 (7.2)	51 (10.4)
Years in practice, median [IQR]	10.0 [2.0,21.0]	8.5 [6.0,12.0]	15.0 [10.0, 25.0]
**Survey questions**			
“How many months before the initiation of hemodialysis do you think an AV fistula should be created?” mean (SD)	6 (2)	6 (2)	6 (3)
“At what estimated GFR should an AV fistula be created?” mean (SD)	18.0 (4.0) ml/min/1.73 m^2^	18.6 (3.8) ml/min/1.73 m^2^	17.5 (3.6) ml/min/1.73 m^2^
“Do you think that surgery to create an AV fistula should be done when the likelihood of developing ESRD in the next year is at or above”, % mean (SD)	67 (14)%	66 (19)%	68 (17) %

### Outcomes after preemptive vascular access placement

Among the 116 patients who underwent preemptive vascular access creation at UCSF, the mean age was 64.3 (±14.5) and the mean estimated GFR was 16.1 (±6.8) ml/min/1.73 m^2^ at the time of access creation (Table 2). The vast majority of the patients underwent surgery for AV fistula (N = 109; 94%) with the remainder undergoing surgery for AV graft. In the year after surgery, only 57 out of the 116 patients (49.1%) patients initiated maintenance HD (Figure 1). There were no deaths. Mean time from access placement to ESRD was 5 (±4) months. Patients who initiated HD within 1 year were more likely to have coronary heart disease (47% vs. 22%; p = 0.005) and had lower estimated GFR level at the time of vascular access construction (13.8 vs. 18.6 ml/min/1.73 m^2^; p < 0.001). There were no other obvious differences in co-morbid conditions or etiology of CKD.

**Table 2:** Baseline characteristics of patients with preemptive vascular access placement at the time of access construction at University of California, San Francisco (N = 116)

**Characteristic**	
Age, mean (SD), y	64.3 (14.5)
Male (%)	48.3
Race (%)	
White	30.2
Black	24.1
Asian	36.2
Hispanics	5.2
Other	4.3
Estimated GFR, mean (SD), ml/min/1.73 m^2^ *	16.1 (6.8)
AV Fistula (%)	94.0
AV Graft (%)	6.0

*calculated by the MDRD equation.

### Probability of ESRD according to prediction equation

Among the 116 patients at UCSF who underwent AV access surgery, the Tangri risk score could be calculated only for 67 patients, as the rest never had their urine albuminuria quantified (most had urine total protein quantified instead). The mean and median time between vascular access surgery and measurement of urine albuminuria was 11.2 (17.9) months and 3.3 (IQR 1.3-13.4) months, respectively, among the 67 patients. The calculated *2-year* probability of ESRD was 61 ± 29% for patients who in fact did develop ESRD within 1 year (N = 37) and 33 ± 21% for the patients who did not develop ESRD within 1 year (N = 30) (p < 0.001) (Table 3). Very similar results were seen when we limit the comparison to the 49 patients who have urine albumin quantified within a year of the AV access surgery (also 61% vs. 34%, p < 0.001) (Table 3).

**Table 3:** Predicted risk of ESRD among patients who did undergo pre-emptive AV access surgery

		**Hemodialysis within 1-year of AV access surgery**	
		**Yes**	**No**
		**N = 37**	**N = 30**
2-year risk of ESRD by Tangri equation in all patients with measured albuminuria (N = 67)	Mean ± SD	61% ± 29%	33% ± 21%
	Median (IQR)	66% (40% –83%)	28% (19% – 39%)
2-year risk of ESRD by Tangri equation in patients with albuminuria measured within 1 year of vascular access surgery (N = 49)		N = 26	N = 23
	Mean ± SD	61% ± 30%	34% ± 22%
	Median (IQR)	65% (40%-84%)	28% (19%-38%)

## Discussion

Our study contributes novel information to the literature by quantifying the targets and expectations of practicing nephrologists with regard to timing of preemptive AV access surgery. Respondents to our surveys reveal that they anticipate an estimated GFR of around 18 ml/min/1.73 m^2^ is about 6 months prior to start of ESRD or when the likelihood of ESRD within the next year is more than two-thirds. We believe these data help us understand the observed current preemptive AV access surgery practice patterns—and why numerous patients do not start dialysis within 12 months after surgery.

To our knowledge, prior to 2011, only two papers examined rates of ESRD after preemptive creation of dialysisAV access. O’Hare et al. investigated only U.S. military veterans [12] and Weber et al. only Canadian patients [13]. Since we initiated our study, two more relevant papers have been published. Kimball et al. investigated outcomes among two academic centers in the U.S. (Denver and Portland) [14] and Oliver et al. leveraged a province-wide data base in Ontario, Canada [15]. Results across these four published studies (O’Hare [12], Weber [13], Kimball [14], Oliver [15]) and ours are quite similar. The rate of initiating dialysis one year after preemptive AV access surgery was 57% in O’Hare [12], 57% in Oliver [15], and 49% at UCSF Medical Center (53% if PD were counted). The mean estimated GFR at time of preemptive AV access placement was 16.1 ml/min/1.73 m^2^ in our study and 17.7 ml/min/1.73 m^2^ in the O’Hare study [12]. In the Kimball paper, 108 out of 150 of their patients had stage 4 CKD at time of surgery (i.e. estimated GFR above 15 ml/min/1.73 m^2^). The study by Oliver et al. [15] did not have information about estimated GFR at the time of surgery. Estimated GFR at time of surgery was lower at 12 ml/min/1.73 m^2^ in the Canadian study by Weber et al. and that study had a higher rate of start of dialysis initiation after surgery (although their data were not presented in a directly comparable manner).

Of note, the observed estimated GFR in the U.S. studies—O’Hare, Kimball and ours--matches quite well with what 3 different groups of U.S. nephrologists report their target estimated GFR to be at the time of AV fistula creation (~18 ml/min/1.73 m^2^) (Table 1).

One hypothesis which can explain these findings is that many U.S. nephrologists do not have a good appreciation of the natural history of disease in advanced CKD. We consistently overestimate the estimated GFR level at which dialysis is imminent. The goals of placing preemptive AF fistulas as expressed by the nephrologists are to have this procedure done about 6 months of starting dialysis, or when the risk of ESRD is judged to be two-third or more likely in the next year. That the relatively high estimated GFR selected is not consistent with these targets is illustrated by the few natural history studies of advanced CKD that exists. Levin et al. followed all CKD patient referred to nephrology in the province of British Columbia who had consistently low estimated GFR. Among those with estimated GFR 15–24 ml/min (N = 1905, mean age 68 years old), the cumulative risk of ESRD (receipt of dialysis or transplant) was only 11% after one year [16]. Among those with estimated GFR < 15 (N = 647, mean age 67 y.o.) the cumulative ESRD risk was only 32% after one year [16]. Similarly, O’Hare documented in a comprehensive national sample of U.S. veterans, that among in the 65–74 years old group, for those with estimated GFR 15–29 ml/min/1.73 m^2^, the one-year risk of ESRD was only 9%. And even for those with estimated GFR < 15 ml/min/1.73 m^2^, the one-year risk of ESRD was only 51% (these rates do vary by age) [17].

Guideline writers and nephrologists may not appreciate that the rate of loss of estimated GFR in advanced CKD can often be very slow. In both the Levin and the O’Hare studies, the median rate of decline of estimated GFR in advanced CKD on the order of 2–3 ml/min/1.73 m^2^ per year [12,16]. Elderly patients in particular have on average slower progression by creatinine based estimated GFR. Furthermore, many patients remain with stable (or even improving) kidney function for months to years without progression. Frequent non-linear estimated GFR trajectory and non-progression has now clearly been documented also at earlier stages of CKD [18].

We speculate that the targeting of a higher estimated GFR threshold then that which matched anticipated time to start of dialysis may have been influenced by the wide promulgation of the NKF KDOQI CKD guidelines. Since stage 5 CKD is labeled as “kidney failure,” some may conflate estimated GFR of 15 ml/min/1.73 m^2^ as being equivalent to imminent need for dialysis. This notion may have been enforced by high profile studies which designate estimated GFR < 15 ml/min/1.73 m^2^ without dialysis as being “untreated” “kidney failure” [19], although recent initiatives by KDIGO have attempted to distinguish between stage 5 requring dialysis versus stage 5 disease not requiring dialysis [20].

While one can debate whether or not having approximately half of the patients starting dialysis within one year after AV access surgery is a high or low percentage, this discussion does not diminish the conclusion that nephrologists’ stated goals are internally inconsistent.

Unfortunately, it does not appear likely that the currently best available prediction equation regarding risk of ESRD will be directly helpful in guiding clinical decision making. While the Tangri equation has some discriminating power (i.e. assessed 2-year risk of ESRD to patients who did go on to receive hemodialysis within a year is higher than the assessed risk for those who did not), the absolute predicted risks are quite far from observed risks (the two groups in Table 3 have actual observed 1-year risks of 100% and 0%). This suboptimal performance means that the equation cannot be introduced into clinical practice.

We believe that our results and the prior literature point out the need for more studies to better understand the natural history of advanced CKD. Unlike during earlier phases of CKD, when management is unlike to change substantially when GFR declines by 5 or 10 ml/min/1.7 m^2^, during advanced CKD nephrologists must make many important decisions over a relatively narrow range of GFR. Missing the window for AV fistula creation is certainly undesirable and this has been much emphasized in the literature. Although this “window” may be later than what has been generally practiced as earlier hemodialysis start has not been associated with significant improvement in cardiovascular events or survival [21,22]. Excessively early placement is also not optimal since surgery incurs pain and discomfort and risk of steal syndrome, high output heart failure and other problems. Some patients with advanced CKD would die without needing HD and would have been better off not undergoing preemptive vascular access surgery. A better understanding of the natural history of CKD in generalizable populations, with emphasis on understanding difference within particular patient subgroups, will be key to improve practice patterns and patient outcomes.

Our study had several strengths. We were able to study the natural history of CKD from both the patient and provider perspectives, which revealed inconsistencies in the minds of nephrologists on timing of access placement in relation to the natural history of progression of advanced CKD. Detailed chart review was performed consistently by a single senior nephrology fellow. Our study had some limitations as well. Our survey sample size was small and our survey was only administered to physicians practicing in California. Our study focused on eGFR thresholds for referral to vascular access placement, however we acknowledge that proteinuria is a very important determinant of renal prognosis. Our chart review study was based on only one academic medical center and not large. In a supplementary analysis, we also reviewed the charts of all consecutive patients seen in the San Francisco Veterans Affairs Medical Center Nephrology clinic between July 1, 2009 and June 30, 2010 (N = 199) and found similar results. Over this time period, 20 patients underwent preemptive vascular access creation (at mean estimated GFR 21 ml/min/1.73 m^2^). Among them, only 7 (35%) proceeded to HD within one year of vascular access creation. One patient died prior to needing dialysis. More importantly, as described above, the estimated GFR at the time of AV access surgery and the rates of ESRD following surgery we noted are similar to findings from other studies in diverse settings.

## Conclusions

In conclusion, while opinion leaders continue to advocate placement of AV fistulae at 20 ml/min/1.73 m^2^[23], based on everything we know so far, using this threshold will result in surgery in many patients possibly years before start of HD and will lead to unnecessary surgery procedures in numerous patients who never progress to ESRD. A more personalized approach is needed.

## Abbreviations

AV: Ateriovenous; CKD: Chronic kidney disease; ESRD: End-stage renal disease; HD: Hemodialysis.

## Competing interest

The authors declare that they have no competing interest.

## Authors’ contributions

NB was responsible for study design, data analysis and manuscript preparation, CH was responsible for data collection and manuscript preparation, DM was responsible for data collection, KLJ assisted in data collection and preparation of manuscript and CYH was responsible for study design, data oversight and manuscript preparation. All authors read and approved the final manuscript.

## Pre-publication history

The pre-publication history for this paper can be accessed here:

http://www.biomedcentral.com/1471-2369/14/115/prepub

## Supplementary Material

Additional file 1: Appendix Physician Survey. Click here for file
