# Supplementary material for: Timing of preemptive vascular access placement: do we understand the natural history of advanced CKD?: an observational study
Source: BMC Nephrol. 2013 May 28;14:115. doi: 10.1186/1471-2369-14-115 (PMC3671964; doi:10.1186/1471-2369-14-115)
Supplement: Additional file 1: Appendix — Physician Survey. [file 1471-2369-14-115-S1.docx]

**APPENDIX**

1. How many months before the initiation of hemodialysis do you think an AV fistula should be created? ____ months

or __ I do not consider this parameter in my decision-making

2. At what estimated GFR should an AV fistula be created? 5, 10, 15, 20, 25, 30 mL/min/1.73m^2^, or other ___

or __ I do not consider this parameter in my decision-making

3. Do you think that surgery to create an AV fistula should be done when the likelihood of developing ESRD in the next year is at or above: 10%, 20%, 30%, 40%, 50%, 60%, 70%, 80%, 90%? (please chose one threshold only)

or __ I do not consider this parameter in my decision-making

4. What criteria/factors do you use to decide when to refer a patient for AV fistula creation?

________________________________________________________________________________________________________________________________________________________________________________________________________________________________________________________________________________________________________________________

5. What is your age? _________ years

6. How many years has it been since you completed your nephrology fellowship? ________ years

7. In what city, state/province/etc., and country do you practice? ______________________________________________________________________________

8. What type of medical practice do you work in? Solo private practice, group private practice, hospital owned practice, HMO, academic, or other:____________________________________
